# An Improved Algorithm to Generate a Wi-Fi Fingerprint Database for Indoor Positioning

**DOI:** 10.3390/s130811085

**Published:** 2013-08-21

**Authors:** Lina Chen, Binghao Li, Kai Zhao, Chris Rizos, Zhengqi Zheng

**Affiliations:** 1 College of Information Science and Technology, East China Normal University, Dongchuang Road 500, Shanghai 200241, China; E-Mail: zqzheng@ee.ecnu.edu.cn; 2 College of Mathematics, Physics and Information Engineering, Zhejiang Normal University, Yingbin Road 688, Jinhua 321004, China; 3 School of Surveying and Geospatial Engineering, University of New South Wales, Sydney 2052, Australia; E-Mails: binghao.li@unsw.edu.au (B.L.); kai.zhao@unsw.edu.au (K.Z.); c.rizos@unsw.edu.au (C.R.)

**Keywords:** double-peak Gaussian distribution, kurtosis testing, location fingerprinting, indoor positioning

## Abstract

The major problem of Wi-Fi fingerprint-based positioning technology is the signal strength fingerprint database creation and maintenance. The significant temporal variation of received signal strength (RSS) is the main factor responsible for the positioning error. A probabilistic approach can be used, but the RSS distribution is required. The Gaussian distribution or an empirically-derived distribution (histogram) is typically used. However, these distributions are either not always correct or require a large amount of data for each reference point. Double peaks of the RSS distribution have been observed in experiments at some reference points. In this paper a new algorithm based on an improved double-peak Gaussian distribution is proposed. Kurtosis testing is used to decide if this new distribution, or the normal Gaussian distribution, should be applied. Test results show that the proposed algorithm can significantly improve the positioning accuracy, as well as reduce the workload of the off-line data training phase.

## 1. Introduction

One of the key issues for location based services (LBS) is the positioning technology, and the indoor positioning accuracy requirement is usually higher than that for outdoors [1,2]. For outdoor environments, a Global Navigation Satellite System (GNSS) such as Global Positioning System (GPS) is ideal. However, GPS is not suitable for indoor environments as the satellite signals cannot penetrate walls or roof of buildings [3,4]. Furthermore, when assisted-GPS techniques are used, the position may have errors of tens of meters [5].

Wi-Fi has been widely used for indoor positioning. Wi-Fi provides local wireless access to a fixed network that is low cost, widely deployed and whose indoor coverage is still rapidly increasing. Using existing infrastructure for positioning is a very attractive option. However, only Wi-Fi signal strength (SS) measurements by the user receiver device from the access points (AP) are available for positioning.

One obvious approach is to convert the SSs to distance measurements. If three distances between the user receiver and different APs can be obtained, trilateration can be used to estimate the receiver's position. However creating an accurate model to convert SS to distance is difficult. The propagation of radio signals in indoor environments is very complicated. The SS received from an AP varies significantly (up to 15 dBm) over time at the same location. In addition, indoor environments vary considerably from each other, which means one model may work well for a specific environment, but perform poorly in other situations. Hence it is difficult, if not impossible, to accurately obtain distance measurements from SSs on a consistent basis [6]. On the other hand, the so-called “fingerprinting” approach has demonstrated promising performance of Wi-Fi positioning [7,8]. There are many advantages of the fingerprinting approach [9,10], including the fact that no special hardware is required on the user mobile station (MS) side [11,12].

However, there is a major shortcoming of this technique—initial creation of the fingerprint database and maintaining it are not trivial tasks. An entry in the fingerprint database is represented by a location identifier paired with the Wi-Fi received SS (RSS) at that location. The “fingerprints” may be the average RSSs (deterministic approach) [7], or RSS distributions (probabilistic approach) [8]. The more accurate the fingerprint database (also referred to as the “radio map”) created, the better the positioning accuracy that can be achieved.

The probabilistic approach can provide better accuracy for Wi-Fi positioning. However, understanding the statistical characteristics of the RSSs is the key [12]. In [13] a lognormal distribution was used to model the RSSs. A shape-filtered empirical distribution was utilised to estimate the RSS distribution [14]. Kamol *et al.* investigated the use of a Gaussian distribution to approximate the real RSS distribution [11]. The Weibull function for approximating the Bluetooth RSS distribution was discussed in [15]. Although the normal distribution is often used for Wi-Fi RSS [16,17], the current study has found that it is not always correct.

This paper proposes a new algorithm based on an improved double-peak Gaussian distribution (IDGD) for Wi-Fi fingerprinting. The rest of the paper is organised as follows: Section 2 gives some details of the probabilistic approach of fingerprinting technology and discusses the characteristics of RSS. Section 3 introduces the IDGD and describes a way to generate the fingerprint database. Before the conclusions are given in Section 5, testing of the new algorithm and the analyses are reported in Section 4. Results show that the proposed algorithm can improve the positioning accuracy by about 20% and has the potential to significantly reduce the labour costs for the training phase.

## 2. Fingerprinting Technique

**Figure 1.**
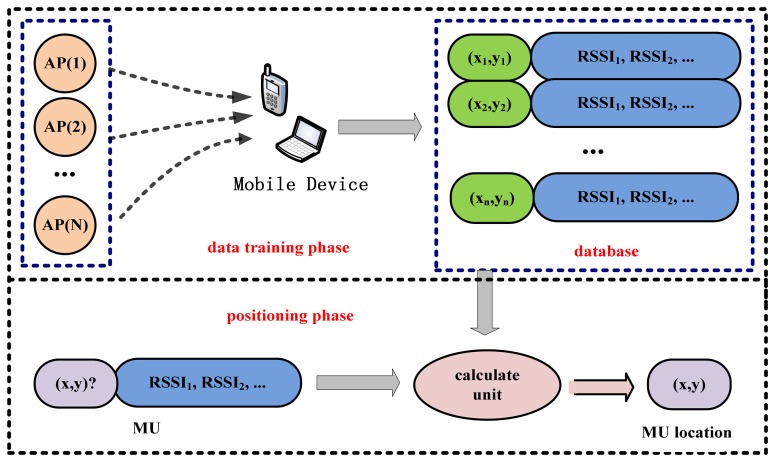
Location fingerprinting technique.

The fingerprinting technique is widely used where line-of-sight signal propagation is not typical. The low cost of the user hardware and the promising performance are its main advantages. Wi-Fi location fingerprinting consists of two phases: the off-line data training phase and the on-line positioning phase. The aim of the training phase is to build a fingerprint database. To generate the database in a conventional way, some reference points (RP) in the area of interest are selected. Locating a MS at one RP location, the RSSs of all the APs are measured. From such measurements the characteristic feature of that RP is determined, which is then recorded in the database. This process is repeated at another RP, and so on until all RPs are visited. In the positioning phase, the MS measures the RSS at a place where it requires its position. The measurements (including RSSs and MAC addresses of the APs) are compared with the data in the database using a matching algorithm. Typically, the signal distance is computed. The smallest signal distance indicates the best match and the likeliest location of the MS can be determined [18,19]. Figure 1 illustrates the whole process.

In this study the authors have adopted the probabilistic approach [20,21]. The location fingerprint is a vector P of the probabilistic RSS values from multiple APs at a particular location L. A typical vector P = (p_1_, p_2_, …, p_N_) consists of N RSS values from N APs. The database contains RSS vectors for all RPs in the area of interest. For positioning, a MS obtains a sample of the RSS vector S = (s_1_, s_2_, …, s_N_). The probability between the P and S for each P in the database is computed. The location is then estimated to be that L for which the probability is the highest. Note that the vector S is random. An error is made when the highest probability occurs for a location L that is not the one at which the sample S was collected. Errors occur because the measured RSS vector is a sample of a random vector while only the probabilistic RSS vector is stored in the database.

### 2.1. Fingerprint Database

The RSS probability distributions of all APs at all RPs need to be stored. The fingerprint of the i-th RPs can be defined as:

(1) $$R_{i}=\left[\begin{array}{cccc} P_{A_{1}}(T_{1}) & P_{A_{2}}(T_{1}) & \cdots & P_{A_{N}}(T_{1})\\ P_{A_{1}}(T_{2}) & P_{A_{2}}(T_{2}) & \cdots & P_{A_{N}}(T_{2})\\ \vdots & \vdots & \vdots & \vdots \\ P_{A_{1}}(T_{M}) & P_{A_{2}}(T_{M}) & \cdots & P_{A_{N}}(T_{M}) \end{array}\right]$$

where A_n_ (n = 1…N) means the n-th AP; T means the measurement of RSS. P is expressed as:

(2) $$P_{A_{n}}(T_{m})=\frac{C_{T_{m}}}{N_{i}}$$

where *N_i_* is the total number of training samples collected at the i-th RP; and $C_{T_{m}}$is the number of *T_m_* appearing in the training data at the i-th RP. The whole fingerprint database is expressed as:

(3) $$D=\left[R_{1},R_{2},\cdots ,R_{w}\right]$$

where w is the total number of RPs in the area of coverage.

To speed up the computations, the signal strength distribution is typically divided into p bins. The fingerprint of the i-th RP also can be expressed as:

(4) $$R_{i}=\left[\begin{array}{cccc} P_{A_{1}}(B_{1}) & P_{A_{2}}(B_{1}) & \cdots & P_{A_{N}}(B_{1})\\ P_{A_{1}}(B_{2}) & P_{A_{2}}(B_{2}) & \cdots & P_{A_{N}}(B_{2})\\ \vdots & \vdots & \vdots & \vdots \\ P_{A_{1}}(B_{P}) & P_{A_{2}}(B_{P}) & \cdots & P_{A_{N}}(B_{P}) \end{array}\right]$$

Correspondingly the probability of the RSS measurements within the bin *B_k_* for AP *A_n_* at the i-th RP can be expressed as:

(5) $$P_{A_{n}}(B_{k})=\frac{C}{N_{i}}$$

where *C* is the number of samples with the signal strength within *B_k_*[15].

### 2.2. The Characteristics of RSSs

**Figure 2.**
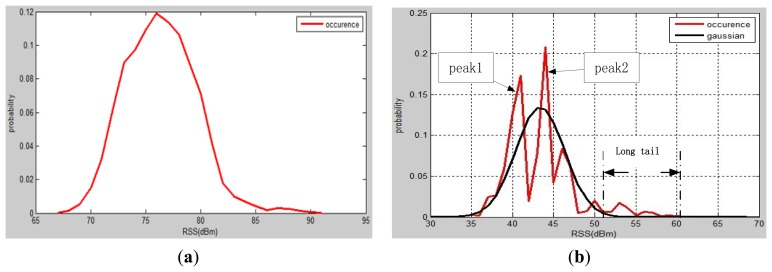
Distribution characteristics of Wi-Fi signals in indoor test environments: (**a**) Guassian; (**b**) Double-peak.

**Figure 3.**
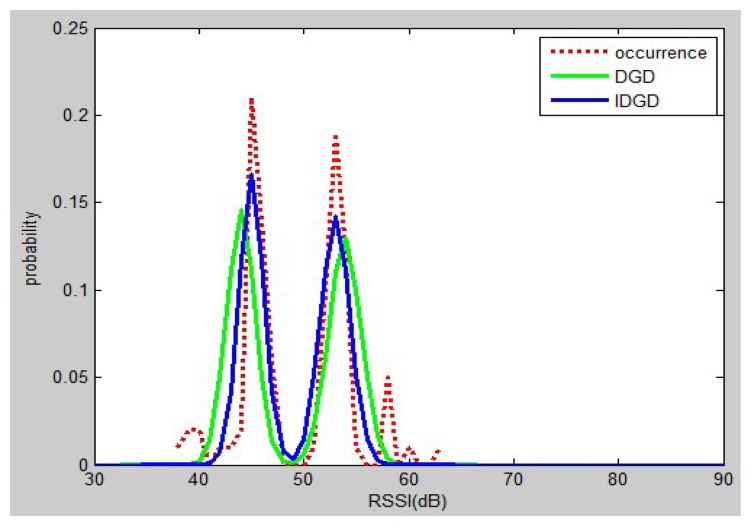
Comparison of DGD and IDGD with the empirical distribution of RSS.

In order to investigate the characteristics of RSSs, Four tests have been carried out at four different environments: a residential room, an office, a class room and a shopping centre. More than 10,000 RSS samples have been collected for each test. In total 424 APs have been detected during the tests. All data have been analysed and some characteristics of the Wi-Fi signals were determined (see Figure 3):

(1) Distribution of more than 30% of RSSs from APs consists of two peaks and a long tail as shown in Figure 2. The two peaks are quite obvious, however this characteristic has not been mentioned in past studies.
(2) The Gaussian function does not approximate the distribution of RSSs very well. The Gaussian distribution in Figure 2 was based on the data used to generate the occurrence plot. Obviously these two distributions are significantly different.

The two peaks distribution of RSS is not accidental. The test results are listed in Table 1. The probability distribution of 134 APs (out of a total of 424) indicate double peaks, which is about 32%. Further investigation has found that the percentage of double-peak distribution of RSS at different environments is not significantly different (being from 26% to 38%), which suggests the double-peak behaviour may not be so unusual for indoor environments.

Generating the database is a prerequisite for location fingerprinting. Generally speaking, the more measurements obtained at each RP, the better the positioning performance. However, more measurements means more effort is required for the RSS survey/training phase. In reality only a few samples of RSS are collected at each RP, and hence the limited samples cannot be used to generate an accurate empirical RSS distribution.

## 3. Improved Double-Peak Gaussian Distribution (IDGD)

### 3.1. Fingerprint Database Based on Gaussian and Double-Peak Gaussian Distribution

The Gauss function is a traditional method for fingerprint database generation [22]; its probability density function can be expressed as:

(6) $$\begin{array}{cc} F(x)=\frac{1}{\sqrt{2\pi \sigma }}e-\frac{{\left(x-u\right)}^{2}}{2\sigma^{2}} & \left(\sigma >0\right) \end{array}$$

where x is the variable of the function; u is the mean of x; and σ is the standard deviation of x. Since the variation of the RSS at each point is large, the probabilistic approach in principle can achieve more accurate results. The probabilistic approach to date has been based on the assumption of a Gaussian distribution for the RSS values. Unfortunately, the distribution of the RSS is not always Gaussian (as mentioned in the previous section).

The double-peak Gaussian distribution (DGD) is proposed as a candidate to replace the Gaussian distribution when it is not suitable. The RSS of each AP is divided into two parts, according to the minimum value between the two peaks, and each part is treated as an independent Gaussian function. The weight of each function was assumed to be 1/2. Its probability density is expressed as:

(7) $$\begin{array}{cc} F(x)=\frac{1}{2}\left(\begin{array}{l} \frac{1}{\sqrt{2\pi }\sigma_{1}}e-\frac{{\left(x-u_{1}\right)}^{2}}{2{\sigma_{1}}^{2}}+\\ \frac{1}{\sqrt{2\pi }\sigma_{2}}e-\frac{{\left(x-u_{2}\right)}^{2}}{2{\sigma_{2}}^{2}} \end{array}\right) & \left(\sigma >0\right) \end{array}$$

where *u_1_*, *σ_1_* and *u_2_*, *σ_2_* are the mean and the standard deviation of the RSS in part one and part two, respectively. However, another problem was observed during the test—the mean values (*u_1_* and *u_2_*) usually were not coincident with the values of the peaks. These offsets introduce some errors in the positioning phase.

### 3.2. IDGD Fingerprint Database Model

In order to solve the problem mentioned above, an improved DGD was developed. The *u_1_* and *u_2_* are changed from the mean values to the values of peak 1 and peak 2, and the standard deviations are the same as *σ_1_*, *σ_2_* used in DGD. Figure 3 shows the empirical distribution, DGD and IDGD.

It can be seen that the probability distribution based on with the IDGD is better than that obtained using the DGD. As already mentioned, the distribution of RSSs is not always Gaussian, but it is also not always double-peak Gaussian. Hence, a new model (IDGD), comprising a joint model of Gaussian distribution and DGD, is proposed. The function is defined as:

(8) $$F(x)=\{\begin{array}{l} \begin{array}{ll} \frac{1}{\sqrt{2\pi }\sigma }e-\frac{{\left(x-u\right)}^{2}}{2\sigma^{2}} & ;\text{one peak} \end{array}\\ \begin{array}{cc} \frac{1}{2}\left(\begin{array}{l} \frac{1}{\sqrt{2\pi }\sigma_{1}}e-\frac{{\left(x-u_{1}\right)}^{2}}{{2\sigma_{1}}^{2}}+\\ \frac{1}{\sqrt{2\pi }\sigma_{2}}e-\frac{{\left(x-u_{2}\right)}^{2}}{{2\sigma_{2}}^{2}} \end{array}\right) & ;\text{two peak} \end{array} \end{array}(\sigma >0)$$

In Equation (8), the Gaussian model is adopted when the distribution of RSSs has one peak, and the DGD is utilised when the RSS distribution has two peaks. The values at peak 1 and peak 2 are denoted as Max 1 and Max 2, respectively. The values between Max 1 and Max 2 are searched (see Figure 2) and the minimum value is found and denoted as Min. We tested all collected data and thus find that if 2 MIN< Max_1_ + Max_2_, the RSS distribution appears one peak or two unobvious peaks. Otherwise, the distribution appears two peaks. Investigation of this question will be carried out in future research. Thus the decision rule is expressed as:

$$\begin{array}{ccc} If & \text{Min}\geq \frac{{\text{Max}}_{1}+Max_{2}}{2}, & \text{use Gaussian model}\\ If & \text{Min}<\frac{{\text{Max}}_{1}+{\text{Max}}_{2}}{2}, & \text{use IDGD model} \end{array}$$

where *u* and *σ* are the mean and standard deviation of the RSS measurement for the Gaussian model. If the IDGD model is used, the RSS measurement is divided into two parts by the mean first. *u_1_*, *σ_1_* and *u_1_*, *σ_2_* are the improved means and standard deviations of the two parts of the RSS measurement.

### 3.3. The Positioning Procedure

The procedure for fingerprint-based positioning using the proposed joint model is as follows, where steps 1 to 4 are the off-line data training phase and steps 5 to 7 are the on-line positioning phase:

Step1: Choose the RPs, and then collect the RSSs from all APs at each RP.
Step2: Detect the gross errors and filter them out.
Step3: Use a global search procedure to find the two peaks and the minimum value between the two peaks. The two times minimum is compared with the sum of the two peaks to decide between using the Gaussian model or the alternative model. This decision rule was created based on all the data collected.
Step4: Create the fingerprint database.
Step5: RSSs are collected by the user, outliers are removed. Calculate the probability distribution of received RSSs.
Step6: Use the fingerprint database to calculate the joint probability density for the RSSs collected in the step 5.
Step7: Estimate the user's location using the K weighted nearest neighbour (KWNN) algorithm. KWNN is a conventional algorithm used for fingerprint-based Wi-Fi positioning. Using this algorithm, K (K ≥ 2) nearest neighbours (those with the shortest signal distance) of a test vector are chosen. The weighted average of the co-ordinates of K points can be used as the estimate of the user's location. The inverse of the signal distance defines the weight [23].

**Figure 4.**
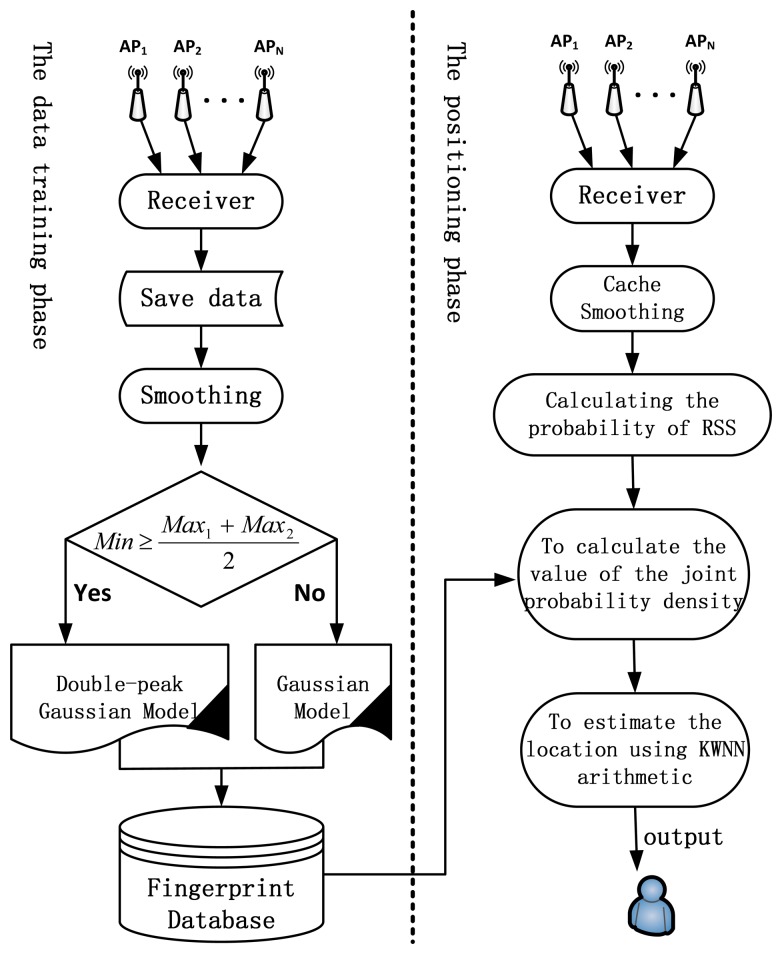
The procedure of positioning using proposed model.

Figure 4 illustrates the details of the procedure using the proposed joint Gaussian and IDGD model for positioning.

## 4. Test and Analysis

To verify the proposed approach, a study was carried out in a small test area. The test area was a typical office room of forty five square metres in size. Nine RPs and five test points (TPs) were selected. A LENOVE X220 Tablet equipped with an Intel Centrino Advanced-N 6250 wireless network card was used to make RSS measurements. A software called inSSIDer was used to collect Wi-Fi signal strengths.

Data were collected during a working day from 8–9 a.m. Up to 100 RSS samples (for about 2 min) were collected at each RP. Then different models—Gaussian, histogram, DGD and IDGD—were used to generate the fingerprint database. About 10–20 RSS samples were collected at each TP soon after the training data were collected. The conventional KWNN (K = 3) was used to estimate the position of the TPs.

In this paper, the weight was calculated as the inverse of the signal distance—the Euclidean distance was adopted. Table 2 lists the positioning error for each TP using different models, and shows that the performance of the Gaussian model is the worst (with an average error of 2.23 m), while using the DGD and histogram generates similar results (1.69 m and 1.73 m, respectively), and the IDGD gives the lowest positioning error. For all individual TPs the performance of the IDGD is almost always the best, and overall performance is improved by about 40%, 20% and 21% compared with those based on the Gaussian, histogram and DGD, respectively. We also try a deterministic approach using the average of RSS for the same experiment, the results are no better (see the first line in the Table 2).

**Figure 5.**
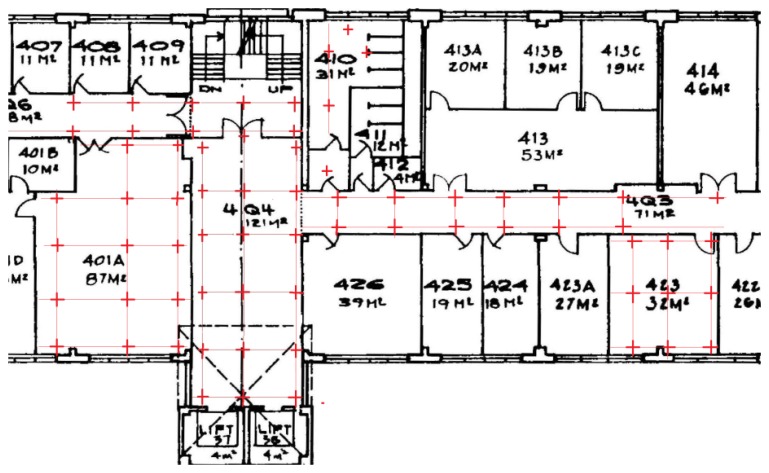
The test bed of the second test.

**Figure 6.**
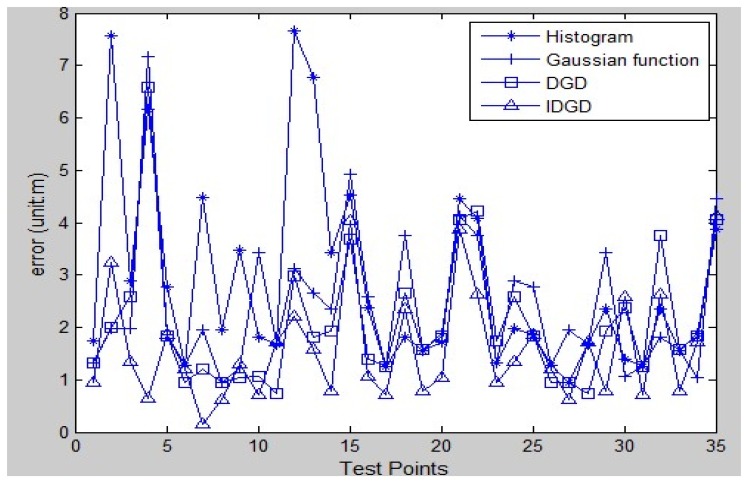
Errors at test points in test 2.

This first test indicated that the proposed model works well. Figure 5 shows the test bed (with an area of approximately 400 square metres) of the second test, consisting of a computer lab, corridors, a foyer, a kitchen and a toilet. In total, there were 68 RPs (red crosses) and 35 TPs, the latter being chosen at random. A similar procedure to the first test was used; there were about 40 RSS samples collected at each RP and 5–20 RSS measurements at each TP. All the data were collected at one working day. Figure 6 shows the results of the test—the horizontal axis is the number of the TP and the vertical axis is the positioning error. In generally the IDGD gave the most accurate positioning results.

**Figure 7.**
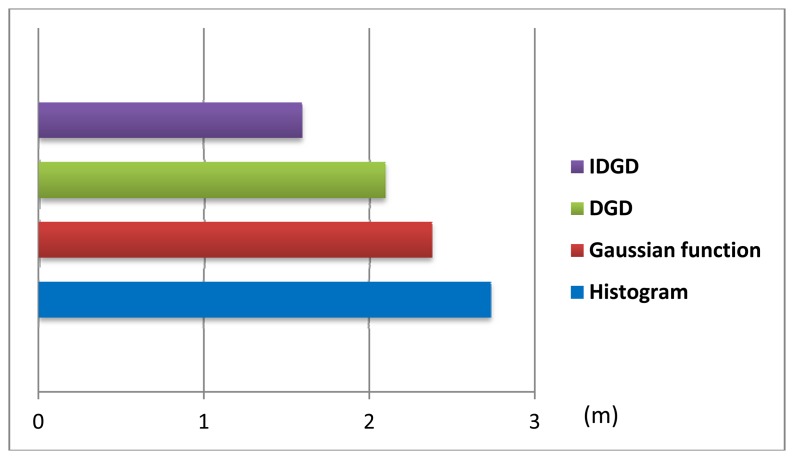
The average error using different models in test 2.

Figure 7 shows the average positioning errors using the four models. The positioning accuracy using the IDGD is improved by about 42%, 33% and 24% compared to that of the histogram, Gaussian and DGD distributions, respectively. The small number of samples collected at each RP is the main reason that the histogram model performed the worst.

## 5. Concluding Remarks

The observation of double peaks of Wi-Fi signal strength has suggested the investigation of a new model known as the Double-peak Gaussian Distribution (DGD) to approximate the signal strength's distribution. Further investigation indicated that an improvement of the DGD was needed, and the Improved DGD (IDGD) was proposed.

The IDGD takes into account the different types of distributions of the RSS samples (sometimes one peak, in other circumstances two peaks). When one peak is detected a standard Gaussian distribution is used to create the fingerprint, whereas when two peaks are detected the DGD is used instead. Tests show that applying the new model for fingerprint-based positioning can significantly improve the positioning accuracy (by up to 40%). Furthermore, this model has the potential to reduce labour costs for the data training phase, *i.e.*, to achieve the same level of positioning accuracy less RSS samples need to be collected during the training phase.

## Figures and Tables

**Table 1.** Double-peak distribution of RSSs in tests

	**The Area of the Test Site**	**The Number of APs Detected**	**The Number of APs Show Double-Peak Distribution**	**Percentage**
Residential room	10 m^2^	28	9	32%
Office	45 m^2^	134	35	26%
Class room	200 m^2^	124	38	31%
Shopping centre	1,000 m^2^	138	52	38%
Total		424	134	32%

**Table 2.** Positioning errors of test 1 (unit: metres).

**Model**	**T1**	**T2**	**T3**	**T4**	**T5**	**Average**
Deterministic	2.28	2.10	0.62	1.27	2.49	1.75
Gaussian	2.36	2.01	1.36	2.57	2.83	2.23
Histogram	2.51	1.27	1.33	1.04	2.28	1.69
DGD	1.26	1.20	1.32	2.58	2.29	1.73
IDGD	1.15	1.23	1.09	1.14	2.18	1.36
